# Examination of three-factor eating questionnaire subscale scores on weight loss and weight loss maintenance in a clinical intervention

**DOI:** 10.1186/s40359-022-00806-8

**Published:** 2022-04-15

**Authors:** Natalie M. Papini, Rachel N. S. Foster, Nanette V. Lopez, Lauren T. Ptomey, Stephen D. Herrmann, Joseph E. Donnelly

**Affiliations:** 1 Department of Health Sciences, Northern Arizona University, 1100 S. Beaver St., Flagstaff, AZ 86001 USA; 2 Department of Internal Medicine, Center for Physical Activity and Weight Management, University of Kansas Medical Center, Kansas City, USA

**Keywords:** Eating behaviors, Restraint, Hunger, Disinhibition

## Abstract

**Background:**

The purpose of this study is to examine three-factor eating questionnaire (TFEQ) scores at baseline and post-intervention (6 months) on successful weight loss and weight maintenance in an 18-month behavioral weight management intervention for adults with overweight and obesity.

**Methods:**

TFEQ and weight were assessed at baseline, 6, and 18 months. Logistic regression models were used to examine scores at baseline on disinhibition, restraint, and perceived hunger factors in the TFEQ on 5% body weight loss at 6 months and 6-month scores to predict 5% weight maintenance at 18 months while controlling for age, sex, and baseline weight.

**Results:**

Participants (n = 287; age = 43.8 ± 10.36 years; female = 64.1%; weight = 222.5 ± 39.02 pounds; BMI = 34.73 ± 4.56) were included for analysis. Dietary restraint at baseline was the only significant predictor of 5% weight loss at 6 months. None of the TFEQ subscale scores at 6 months predicted 5% weight maintenance at 18 months. The model examining weight loss at 6 months accounted for 7% of the variance of the outcome and 11% of the variance of weight maintenance at 18 months.

**Conclusion:**

Dietary restraint is a unique eating behavior associated with weight loss at 6 months beyond other eating behaviors measured by the TFEQ in an adult sample enrolled in a weight loss intervention. No other subscale scores were significant at 6 months or at 18 months. Future research should consider how to promote flexible control and discourage adoption of rigid restraint behaviors since the latter is associated with disordered eating patterns.

## Background

The rising trend in the incidence of obesity is a severe public health issue. A recent study by Wang et al. [1] suggests 78% of American adults will be overweight or obese by the year 2030, a statistic that has been increasing since 1999. In addition, researchers note sex, region, and socioeconomic status all contribute to differences in instances of obesity and overweight throughout the U.S. In 2017, 9.2% of adults in the U.S. were severely obese, with prevalence higher in women (11.5%) compared to men (6.9%) [2]. However, the obesity crisis does not impact the U.S. alone; the World Health Organization notes that in 2016, about 13% of the adult population in the world was obese, a prevalence that increased from 8.7% in 2000 [3]. With the obesity epidemic continuing to grow, health issues associated with this disease are of concern. Obesity is associated with several preventable chronic conditions, including heart disease, cancer, diabetes, and stroke [4]. Despite the fact that health risks associated with obesity and overweight have been largely well-documented, many individuals struggle with participation in traditional weight management programs, with attrition rates up to 53.9% or higher [5]. Additionally, about one-third to two-thirds of dieters regain more weight than they lose, highlighting the need to consider alternative, nondieting approaches and establish effective treatments [6]. In an effort to prevent increases in obesity and related comorbidities, clinical researchers have explored various methods to help achieve meaningful weight loss for these individuals.

Typical multi-component weight management programs, as recommended by current weight management guidelines from the American Heart Association (AHA)/American College of Cardiology (ACC)/The Obesity Society (TOS) target behavior change strategies such as portion control, calorie restriction, lifestyle modifications (increased exercise, reduced fast food consumption), and education/behavioral counseling [7, 8]. These tactics often lead to successful treatment outcomes as defined by ≥ 5% weight loss with maximum weight loss achieved at about 6 months [8–10]. However, only about 20% of individuals with obesity are able to maintain these results after treatment [11]. Although scientists have identified successful interventions, research is still in a nascent stage in understanding specifically which aspects of treatment work in creating a successful weight maintenance and behavior-change program tailored to individual needs [12, 13].

One clinically validated psychometric scale that quantifies specific dimensions of eating behavior is the Three Factor Eating Questionnaire (TFEQ). The TFEQ measures cognitive control “restraint” of eating behavior (Factor I, TFEQ-R), disinhibition of control (Factor II, TFEQ-D), and perceived hunger (Factor III, TFEQ-H). Restraint refers to one’s ability to restrain food intake, such as utilizing portion control and avoiding high calorie foods. Disinhibition refers to overeating food in response to various stimuli associated with losing control of food intake and eating opportunistically, for example, eating in stressful situations. Hunger refers to how the feeling of being hungry modulates food intake, for example, feelings of hunger resulting in mass quantities of food consumption [14]. The TFEQ is popular due to its utility in measuring factors with direct clinical relevancy, examining behaviors related to both eating styles and personality traits [15].

Recent research has examined the relation between TFEQ factors and obesity, indicating mixed findings [16–18]. Some research suggests that TFEQ-R has a positive relation with BMI, such that higher TFEQ-R scores predict obesity; however, other research notes that TFEQ-R scores and BMI have an inverse relation, a relation that makes most sense theoretically [17, 18]. Since restraint refers to an individual’s ability to restrain themselves from making impulsive food choices, one would expect an individual to make better health-based decisions and thus have a lower BMI if they were more apt to demonstrate restraint in their food intake. In a systematic review of the TFEQ literature by Bryant et al. [14], researchers found that higher TFEQ-R scores coincided with lower average energy intake, lower fat intake, and lower appetite ratings.

Bryant et al. [16] reviewed the literature on the effects of TFEQ-D on appetite and weight management. They found that greater TFEQ-D was significantly associated with higher body mass index (BMI), low levels of physical activity, low levels of self-esteem, and predictive of weight regain after weight loss. Interestingly, higher scores on the TFEQ-D subscale and higher scores on the TFEQ-R subscale have been related to greater eating disordered pathological behavior, such that greater disinhibition has been linked to binge eating disorders, and higher restraint has been related to greater disordered eating and less intuitive eating [19–21].

While TFEQ-R and TFEQ-D have received much attention in the weight management literature, TFEQ-H has been less widely researched, perhaps due to its sensitivity to state-based hunger. A study by Yeomans and McCrickerd [22] indicated that acute hunger significantly impacts TFEQ-H scores, with greater state-based hunger relating to higher TFEQ-H scores. Thus, the TFEQ-H subscale may be inadvertently measuring current hunger and may not be accurately measuring hunger as a behavioral trait. Researchers have observed relations between greater TFEQ-H scores and higher BMI, as well as lower scores following pharmacologically aided weight loss [16, 23, 24].

As these three factors represent unique predictors of eating-related behaviors, there is utility in measuring how they modulate successful treatment outcomes in longitudinal weight management programs. Toward this end, researchers sought to examine the potential of all TFEQ factors at baseline as significantly predictive of weight loss success after an intensive diet and exercise intervention, as well as the predictive value of 6-month scores on clinically significant weight maintenance. The purpose of the current study is to conduct a secondary analysis examining TFEQ scores from baseline and 6-month assessments on successful weight loss and weight loss maintenance in a clinical intervention for adults with overweight and obesity.

## Method

Data for this secondary analysis are from a two-arm randomized controlled equivalence trial, conducted from 2008 to 2011, designed to compare identical behavioral weight loss interventions delivered either by traditional face-to-face (FTF) group meetings or by group phone conference calls (phone) over 18 months (6 months weight loss; 12 months maintenance; NCT01095458). A detailed description of the study design, including inclusion/exclusion criteria, and results for the primary outcomes have been published [25, 26]. Briefly, the primary outcome of the trial was to measure differences in weight loss between groups post-intervention at 6-months and during maintenance at 12-months and 18-months. Results indicated no significant differences in weight loss and weight maintenance between phone versus FTF conditions. This secondary analysis measured TFEQ scores at baseline as a predictor of weight loss and TFEQ scores at 6 months as a predictor of weight maintenance, regardless of group condition.

### Participants

Participants (N = 287) were between 18 and 65 years of age (43.8 ± 10.4) with overweight or obesity, and a BMI (kg/m^2^) between 25 and 39.9. Exclusion criteria consisted of unwillingness to be randomized, participation in a research project involving physical activity or weight management in the previous 6 months, reported planned exercise > 500 kcal/week, reported weight change of ± 2.27 kg for 3 months prior to intake, reported pregnancy during the previous 6 months, lactation, or planned pregnancy during the 18-month study, reported serious medical risk (i.e., type 1 diabetes, cancer, recent cardiac event), exhibiting disordered eating symptomatology determined by a score ≥ 20 on the Eating Attitudes Test (EAT-26) at baseline, disproval of physician consent to participate, exhibiting extreme weight control behaviors (i.e., purging or binge eating in response to extreme caloric restriction), currently taking psychotropic medications or actively seeking counseling, adhering to special diets (i.e., Atkins, vegetarian, etc.), or not having access to shopping and meal preparation (i.e., college students on meal plans, individuals in the military) [27].

Participants were recruited using newspaper advertising, email listservs, public service messages, media contacts, word of mouth, and the waiting list for participation in our ongoing University of Kansas Weight Management Project (KWMP). Participants were recruited over the course of ~ 3.5 years. Written informed consent was obtained prior to engaging in any aspect of this trial. Financial compensation ($300 total) was provided for completing outcome assessments. In the original study, approval for the investigation was obtained from the Human Subjects Committee at The University of Kansas and Informed Consent was signed by each individual before any participation in this investigation.

### Three Factor Eating Questionnaire (TFEQ)

The Three Factor Eating Questionnaire (TFEQ) is a 51-item tool administered to examine current dietary practices and measures three different eating behaviors [28]. The TFEQ includes three factors: restraint (21 items), disinhibition (16 items), and perceived hunger (14 items). Additionally, there are sub-categories within each factor to better understand each of these eating behaviors. Item responses on the TFEQ are scored as 0 or 1 and summed. Higher scores indicate higher levels of restrained eating, disinhibited eating, and predisposition to hunger. TFEQ scores were obtained at baseline, 6, and 18 months by trained research assistants who were blind to group assignment. While the authors concede that more recently developed versions of the TFEQ with improved factor structure and internal consistency have been developed since its initial design, the original factors adequately quantify specific eating behaviors of interest for weight loss and weight management. Despite the factor structure of the original TFEQ failing to be replicated by subsequent studies, Bond et al. [29] confirmed the factor structure in a population of Australian undergraduate women and indicated that these three factors could further be subdivided [30, 31].

### Anthropomorphic measurements

Body weight was recorded at baseline, 6, and 18 months using a digital scale accurate to ± 0.2 lbs (Befour Inc Model #PS6600, Saukville, WI). All participants were weighed between the hours of 6 a.m. and 10 a.m. prior to breakfast wearing a standard hospital gown after attempting to void. Health educators measured height using a stadiometer (Model PE-WM-60–84, Perspective Enterprises, Portage MI) and calculated body mass index (kg/m^2^). Additionally, health educators measured waist circumference using the procedures of Callaway et al. [32].

### Intervention

The behavioral weight management intervention used in this study, the KWMP, is grounded in Social Cognitive Theory (SCT) to promote changes in diet and physical activity. SCT is a triadic, dynamic model that posits an individual's behavior is determined by the reciprocal interaction of personal, behavioral, and environmental factors [33]. This program follows the ACC/AHA/TOS guidelines for the management of overweight and obesity, and is also considered a gold standard treatment in that it is aligned with the characteristics of treatment Yanovski (2017) outlined: at least 14 sessions in 6 months of a comprehensive intervention that can be delivered in group or individual sessions by a trained facilitator for 1 year or greater [7, 34]. Randomization occurred sequentially using a closed envelope procedure that was created by a study statistician and concealed from the investigators and data collection staff until after baseline data collection. Sixty-minute group meetings were held either in-person or over the phone, depending on group randomization, and led by trained interventionists (“health educators”). During the meetings, health educators reviewed and discussed self-report data outlining adherence to the diet and exercise protocol. The health educators then implemented a lesson on nutrition, physical activity, or lifestyle modification. Following the lesson, health educators led a group discussion regarding individual progress on diet and exercise goals. Meetings were held weekly during the weight loss phase (0–6 months), twice per month during months 7–9, once per month during months 10–12, and once every other month from 13 to 18 months.

Participants were encouraged to consume portion-controlled meals (“PCMs”, Health Management Resources, Boston, MA) during both the weight loss intervention and throughout the weight maintenance phase. PCMs consist of portion-controlled liquids shakes (e.g. chocolate or vanilla shakes, chicken soup, or hot cereal) and solid entrees (e.g. beef stroganoff, chicken enchiladas, lasagna) that are relatively low in calories. PCMs consist of portion-controlled liquid (shakes) and solid meals (entrees) that are relatively low in calories. Participants in the FTF condition completed PCM order forms the week prior to the group meetings. Participants in the phone condition ordered PCMs at the mid-week check-in with the health educator and received PCMs via ground transportation within 3–4 days.

All participants were asked to provide mid-week and weekly self-report data of physical activity and diet progress by phone, fax, or email during the weight loss phase. During weight maintenance (months 7–18), participants were encouraged to continue to submit weekly compliance records. All participants received identical notebooks that included a basic outline of the weight loss diet plan, recipes, instructions for physical activity, and supplemental materials to the behavioral lessons.

Health educators received 3–4 months of training in the study protocols, had prior experience with weight management, and had advanced degrees in nutrition, exercise physiology, behavioral counseling, or psychology. All sessions in both conditions were audio recorded. Treatment integrity in both conditions was assessed by having another health educator listen to the audiotapes and compare the content of these meetings with a pre-determined checklist of the essential content and structure for meetings.

### Weight loss phase (6 months)

Energy intake was reduced to ~ 1200 to 1500 kcal/day using a combination of commercially available PCMs, fruits and vegetables, and beverages. Participants were instructed to consume a minimum of 3 shakes at ~ 100 kcal each, 2 entrees (140–270 kcal each), and 5, 1-cup servings of fruits or vegetables each day. Non-caloric beverages (e.g., diet soda, coffee sparkling water) were allowed ad libitum. If participants reported being hungry, they were encouraged to consume more fruits and vegetables or PCMs. AHA/ACC/TOS weight management guidelines demonstrate that a 5% initial weight loss is associated with improvements in many health outcomes [7]. During the weight loss phase, PCMs were provided without cost to participants; however, participants bought their own fruits and vegetables each week.

### Weight maintenance phase (7–18 months)

All participants were instructed to consume a weight maintenance diet with an energy intake designed to maintain weight loss using the equation of Mifflin et al. [35]. Energy intake was adjusted as needed based on an individual's weight each week. Participants were provided a meal plan with suggested servings of grains, proteins, fruits, vegetables, dairy, and fats, based on their energy needs and the USDA's 2005 "My Pyramid.” During weight maintenance, participants were encouraged to continue consuming a minimum of 14 PCMs per week and a minimum of 35 fruits and vegetables per week. All foods and beverages were purchased by the participants.

### Data reports from group meetings

Participants recorded the number of PCMs, fruits, and vegetables consumed daily. Data were submitted via toll-free phone, fax, or email to the health educator twice per week during weight loss (mid-week and day of meeting) and weekly during weight maintenance. If data were not received, the health educator attempted to contact the participant for this information. In the phone group, participants used their own scales to provide a self-reported weight while participants in the FTF clinic group weighed on a scale at the clinic site. These weights were used to monitor progress only and were not the weights used for the primary outcome. Changes in medications and adverse events were reported privately to the health educator at FTF clinic meetings. Participants in the phone group were reminded to place an email or call their health educator if they changed medications or experienced any adverse events.

### Statistical analysis

Frequencies and descriptive statistics for the sample were calculated. Six non-conditional logistic regression models were used to examine the relationship among disinhibition, restraint, and perceived hunger factors in the TFEQ (at baseline) with 5% body weight loss at 6 months (outcome 1) and in the TFEQ (at 6-months) with weight loss maintenance at 18 months (maintenance of the original 5% weight loss, outcome 2). Weight loss at 6 months was selected for outcome 1 because participants were measured following the most intensive period of the intervention (i.e., weight loss phase). Two models controlled for age, sex, baseline disordered eating as measured by the 26-item eating attitudes test (EAT-26), and baseline weight. Based on recent findings, the EAT-26 covariate was removed from the model because it is not psychometrically sound when used to identify disordered eating in adults with overweight and obesity [36]. Removal of the baseline EAT-26 scores as a covariate did not affect significance at 6 or 18 months (data not shown).

## Results

After removing individuals with missing covariates or predictors, a total of 287 participants (72.6% of the original sample) were included in the analysis at 6 months (males = 103 and females = 184) with an average age of 45.26 (SD = 9.22) years. The average baseline BMI was 34.73 (SD = 4.56), and the majority of participants were white (80.1%). Table 1 indicates demographic characteristics of participants in this study. After removing missing data at 18 months, 221 participants were included in the analysis. Results of bivariate analyses indicated that participants who completed study measures at 18-months follow-up did not differ significantly on socio-demographic factors of age or sex, and did not differ on baseline weight, compared to those who did not complete study measures at 18-months follow-up (data not shown). Previous findings indicate no demographic differences between individuals assigned to the face to face and those assigned to the phone-based treatment [25].

**Table 1:** Baseline socio-demographic and anthropometrics of participants included in the analysis (n = 287)

Participant characteristic	N	%
Sex		
Male	103	35.9
Female	184	64.1
Race		
White	230	80.1
Black	42	14.6
Asian	5	1.7
Native Hawaiian	0	0
American Indian	4	1.4
Other	4	1.4
Multi-Racial	2	0.7
Ethnicity		
Hispanic or Latino	21	7.3
Not Hispanic or Latino	218	76
Unknown	48	16.7

	Mean	SD
Age (year)	45.26	9.22
BMI	34.73	4.56
Weight at baseline (lbs.)	222.5	39.02

*SD* standard deviation, *BMI* body mass index (kg/m^2^)

Of the 257 participants who lost 5% of weight at 6-months (89% of sample, 63.0% female), mean weight at baseline was 222.19 lb. (SD = 39.46 lb.) and mean weight at 6-months was 189.45 lb. (SD = 35.05 lb.). Mean weight loss was 32.74 lb. (SD = 14.66 lb.). Of the 139 participants who maintained weight loss at 18-months follow-up (62% of sample, 64.7% female), mean weight at 18-months was 187.32 lb. (SD = 35.58 lb.). Mean difference in weight between 6-months and 18-months-follow-up was 9.00 lb. (SD = 11.99 lb.). In addition to weight outcomes, mean values for restraint increased from baseline to 6-months while disinhibition and hunger decreased from baseline to 6-months (see Table 2).

**Table 2:** Mean baseline and 6-month scores for restraint, disinhibition, and hunger

	Baseline (SD)	6-month (SD)
Restraint	8.13 (3.95)	15.0 (3.34)
Disinhibition	7.48 (3.23)	5.90 (3.02)
Hunger	5.31 (3.24)	4.02 (2.78)

Of the three factors examined, dietary restraint at baseline was the only significant predictor of 5% weight loss at 6 months (see Table 3). None of the scores at 6 months predicted weight loss maintenance outcomes at 18 months. The variables included in the model examining 5% weight loss at 6 months accounted for 7% of the variance of the outcome (as expressed by the Nagelkerke pseudo R^2^) (*R*^2^ = 0.073) and 11% of the variance of weight loss maintenance at 18 months (as expressed by the Nagelkerke pseudo R^2^) (*R*^2^ = 0.11).

**Table 3:** Logistic regressions examining associations between Three Factor Eating Questionnaire Factors at baseline and 5% body weight loss at 6 months and Three Factor Eating Questionnaire Factors at 6-months and weight loss maintenance at 18 months (n = 287)

	Odds ratio (95% CI)	
	6 months (5% weight loss)	18 months (5% weight loss maintenance)
Age (years)	0.62 (0.99–1.09)	1.05 (1.01–1.08)
Sex (M = 0, F = 1)	0.57 (0.20–1.65)	0.56 (0.27–1.16)
Baseline weight (lbs)	0.99 (0.98–1.00)	0.99 (0.98–1.00)
Baseline restraint	0.90 (0.81–1.00)	–
Baseline disinhibition	1.04 (0.90–1.21)	–
Baseline hunger	0.99 (0.85–1.16)	–
*Three factor eating questionnaire*		
Restraint (6-months)	–	1.07 (0.98–1.17)
Disinhibition (6-months)	–	0.91 (0.81–1.02)
Hunger (6-months)	–	1.11 (0.94–1.19)

## Discussion

Results of this study suggest that, of the factors measured by the TFEQ, baseline restraint is the only factor associated with clinically significant weight loss at 6-months. This suggests that there is something unique about dietary restraint as an eating behavior that predicts weight loss over and above other eating habits and behaviors as measured by the TFEQ. This study observed no association between scores in the hunger and disinhibition subscales of the TFEQ and weight outcomes at 6 and 18 months in a sample of adults enrolled in a weight management program.

The current findings have similarities and differences with previous studies that utilized the TFEQ to examine eating behavior. Similar to the current study findings, low cognitive restraint scores were predictive of less than 30% excess weight loss at 12 months in individuals who underwent gastric electrical simulation (GES) [37]. However, Alacron Del Agua et al. [37] also observed a significant inverse relationship between the disinhibition subscale of the TFEQ and weight loss outcomes at 12 months, where higher disinhibition was associated with lower weight loss. Perhaps because of differences in treatment modality (GES vs. behavioral weight loss program), the outcomes reported in the current study are dissimilar to those reported in Alacron Del Agua et al. [37] which found lowered disinhibition scores throughout the weight loss phase were predictive of 5% weight loss maintenance. Future work should investigate the impact of weight loss modality (e.g., gastric bypass surgery, behavioral weight loss programs, intermittent fasting) on the predictive utility of restraint, hunger, and disinhibition subscales of the TFEQ for weight loss outcomes.

Previous work aimed at developing behavioral and psychological profiles of successful weight loss maintainers and individuals who experienced weight regain found that restrained eating was the only factor significantly predictive of successful weight loss at 12-months [38]. These findings are somewhat aligned with those found in the current study with a similar sample of adults who sought weight loss treatments. It is imperative to note that although dietary restraint was predictive of weight loss at 12 months, rigid control can also lead to development of eating disorders, such as binge eating [14, 39]. As noted in Polivy and colleagues [40], defining restrained eaters is complex since there are several explanatory factors that instigate restrained eating (e.g., religious motives, dieting based on the assumption it will improve health, dieting for aesthetic reasons, food allergies). Given these differences, it is important to clearly define that restrained eaters in the current study include adults with overweight and obesity who actively restricted calories with the intention of weight loss. Although baseline restraint scores were associated with clinically significant weight loss at 6 months, scores at 6-months were not predictive of weight loss maintenance at 18 months. This is consistent with a review of how measures of dieting and dietary restraint (as measured through the TFEQ) prospectively predict weight change [41]. Findings suggest no evidence that restraint measured through the TFEQ predicted weight loss over time. It is possible that while dietary restraint may yield clinically significant weight loss at 6 months, this effect diminishes over time.

The present study observed inconsistent findings on the predictive utility of disinhibition than those previously reported. Previous work that utilized different weight loss modalities and dietary methods (e.g., Mediterranean diet, high protein and high carbohydrate diet, calorie restricting diets, and lifestyle intervention including dietary advice) resulted in decreases in disinhibition with accompanying weight loss [42–45]. When examining weight maintenance at 12 months, lower scores on disinhibition predicted weight loss in young women [43]. Other research indicates higher disinhibited eating behavior as linked to higher BMI scores in US adults [46]. In the current study, the disinhibition subscale had no significant implications for weight loss at 6 months or weight loss maintenance at 18 months. It is possible certain salient variables were not measured in the current study that could have an impact on both weight status and TFEQ subscale scores, such as sleep quality. For instance, one study showed that disinhibited eating behavior mediated the relationship between sleep quality and weight status in both males and females, implying that improving sleep quality could benefit weight loss efforts by reducing overeating [46]. Given the growing evidence of how sleep can impact health behaviors and diet, future studies should include measures of sleep quality such as the Pittsburgh Sleep Quality Index (PSQI) in studies examining eating behaviors [47].

Future research should examine the specific aspects of TFEQ cognitive restraint subscale and other behavioral components that it may be inadvertently measuring in order to best quantify and understand specific elements contributing to successful weight loss, weight management, and obesity prevention. Although a greater cognitive restraint score may appear to be advantageous for an individual seeking weight loss in the short-term, some research suggests that cognitive restraint has been related to psychopathology and disordered eating. Westenhoefer [48] found that the factor of cognitive restraint is not a homogenous construct and can be quantified by two different characteristics: flexible control and rigid control, mediated by one’s disinhibition.

Westenhoefer [48] found that higher disinhibition, as measured by rigid control, was associated with an “all or nothing eating” approach, including counting calories, frequent dieting, and eating low calorie foods. Lower disinhibition, also known as “flexible” control, includes a more lenient eating approach, and is associated with eating slowly, stopping eating, and taking small helpings of food. Thus, the measurement of cognitive restraint may be picking up on two distinctive behavioral approaches to weight management and perception of food and calories, with rigid control associated with maladaptive dieting strategies and flexible control associated with adaptive and healthier weight management techniques [29].

Furthermore, the findings of the current study point to flexible control changes measured through the disinhibition subscale during weight loss (0–6 months) as unrelated to weight loss maintenance at 18 months. Westenhoefer’s [48] delineation between flexible and rigid control in the TFEQ has been utilized in subsequent studies to determine the impact of these sub-categories on disordered eating. In a sample of female undergraduate students, Stewart and colleagues [49] found that rigid control was significantly associated with eating disorder symptoms, mood disturbances, and over-concern with body shape and size, while flexible control was not associated with any of these behaviors. Additionally, Westenhoefer et al. [50] found that rigid control was associated with higher BMI and more frequent binge eating, while flexible control was associated with lower BMI and less frequent binge eating. While much research suggests that cognitive restraint, specifically rigid control, relates to eating disordered behaviors, the results of this theory are mixed. Interestingly, a study by Linardon and Mitchell [20] found that the subtypes of cognitive restraint—rigid control and flexible control—were both significantly related to disordered eating, body appreciation, and body image concerns, including body checking. However, researchers found that rigid control more strongly predicted over-evaluation of body weight and shape compared to flexible control. Additionally, Masheb and Grilo [51] found that flexible control and rigid control had no relation to binge eating or overeating in patients with binge eating disorder; flexible and rigid control may not contribute to eating disordered behaviors in specific clinical samples. Thus, there needs to be more research on the differences between how rigid control and flexible control modulate specific disordered eating behaviors.

The hunger subscale of the TFEQ was not a significant predictor of weight loss at 6 or 18 months in the current study. These findings are aligned with previous research noting how infrequently the hunger subscale is reported in the literature and how rarely it is associated with weight change [16]. Additionally, an individual’s current hunger and satiety have been shown to impact hunger scores on the TFEQ, such that individuals who report higher levels of current hunger score higher on the hunger subscale [22]. These findings suggest that the hunger subscale of the TFEQ may be a better indicator of current hunger than trait-hunger. Other studies incorporating alternative measures of hunger (such as the Visual Analog Scale) found that hunger decreased during weight loss and that higher baseline levels of reported hunger were associated with less weight loss [52].

The current study has both strengths and limitations. Notable strengths include the length of follow up, sample size, and investigation of the predictive utility of the TFEQ within a sample of both males and females enrolled in a behavioral weight loss treatment program. One limitation of the current study includes no clinical investigation of binge eating disorder (BED). Individuals with BED typically score lower on the EAT-26, despite disordered eating [53]. Furthermore, binge eating behaviors are the most common eating disorder reported in individuals seeking weight loss treatments [54, 55]. Findings indicate BED may impede weight loss efforts. Pacanowski et al. [56] observed weight gain in individuals enrolled in a cognitive behavioral therapy intervention and found that objective binge eating episodes at the start of treatment predicted weight change. The current study would be improved by replacing the EAT-26 with a psychometrically sound instrument that detects disordered eating in larger-bodied people. This is especially important given that less than 6% of individuals with EDs are medically underweight. In addition to this, including an adequate measure of binge eating to utilize as a covariate in the model would improve the utility of the present study findings.

A major limitation of the current study includes the main outcome variables of BMI and weight loss without inclusion of other important health outcome variables, such as health-related quality of life. Other findings show that health programs that emphasize responsibility for health outcomes by changing individual behaviors have been associated with increased weight [57]. The Health at Every Size (HAES) paradigm incorporates a weight-inclusive approach and promotes size acceptance, balanced eating, physical activity to improve quality of life, and respect for all body shapes and sizes [58]. Research utilizing HAES interventions have demonstrated improvements in psychological outcomes, physical activity, cardiovascular status, eating behaviors, and quality of life [59].

Finally, Hart et al. [60] noted the importance of public health research shifting away from body weight toward health behaviors in order to promote inclusivity and minimize weight stigma in research. Future studies including weight or BMI variables should consider how findings and interpretation of findings encompass or continue weight stigma beliefs and should incorporate additional variables that focus on health behaviors rather than weight outcomes. Another limitation of the current study is the lack of information on subtypes of cognitive restraint contributing to participants’ scores. Thus, researchers are not clear as to what specific behaviors—maladaptive or adaptive—contributed to successful weight loss and weight loss maintenance in this population. However, while the weight loss treatment program offered in 2011 (when these data were collected) included a combination of both flexible and rigid control-type strategies to lose weight, the program placed most emphasis on “staying on plan” (eating 1200–1500 kcal/day), a more rigid control technique. Specifically, behavioral lessons included in the weekly group meetings at this time focused mainly on strategies such as portion control (“plate division”) reading food labels, “basic calorie counting”, and calculating energy expenditure. Since 2011, the program has evolved to include lessons in intuitive eating and stress management, places less emphasis on calorie counting, and emphasizes finding balance between indulging in high calorie foods and eating healthfully. Thus, researchers can hypothesize that the cognitive restraint scores for the participants in this study were likely driven by a combination of both rigid and flexible control approaches, with more influence coming from rigid control.

Future research may examine the impact of a diet intervention that focuses on dietary flexibility and attenuation to one’s own hunger and satiety cues as successful weight loss and weight management techniques, rather than fixation on calorie counting and food avoidance. One such approach is intuitive eating, an approach by which cognitive restraint scores have found to be inversely related. Intuitive eating embodies eating behaviors that are initiated and discontinued based only on physiological hunger and satiety signals, as opposed to environmental triggers or eating for emotional reasons [61]. Intuitive eating is considered to be within the realm of adaptive eating behaviors such that individuals who eat intuitively are internally aware of their physiological level of hunger and satiety, which may serve to protect them from eating disorders or generally unhealthy eating habits [61]. This internal awareness property of intuitive eating is demonstrated in young children and is disrupted as an individual ages due to environmental factors (such as parental or self-enforced restrictions on food) [62]. Sustained/long-term intuitive eating has been associated with fewer depressive symptoms, improved body dissatisfaction, and fewer extreme diet behaviors such as taking diet pills and self-induced vomiting in a diverse sample of adolescents followed longitudinally into young adulthood [63]. Intuitive eating may also be an advantageous approach because of its focus on health and personal enjoyment rather than restriction or weight loss [64]. It is possible that interventions focused on improving intuitive eating may encourage flexible restraint and discourage rigid constraint, thus mitigating disordered eating risk.

## Conclusions

In conclusion, while many factors influence weight management, understanding which factors specifically drive eating behavior and decision making at the individual level is imperative to helping people improve dietary quality while also mitigating the risk of disordered eating and adoption of rigid control strategies. Cognitive restraint is a factor that renders more in-depth investigation for this population. Research should seek to address differences in flexible and rigid control based on chosen weight loss modality and consider how to promote flexible control and discourage against adoption of rigid control and its associated disordered eating patterns in weight loss treatments.

## Data Availability

The datasets used and/or analyzed during the current study are available from the corresponding author on reasonable request.

## Abbreviations

ACC: American College of Cardiology
AHA: American Heart Association
BED: Binge eating disorder
BMI: Body mass index
EAT-26: Eating Attitudes Test (26 item)
FTF: Face to face
GES: Gastric electrical simulation
HAES: Health at Every Size
KWMP: Kansas Weight Management Project
PCM: Portion-controlled meal
PSQI: Pittsburgh Sleep Quality Index
SCT: Social Cognitive Theory
TFEQ: Three Factor Eating Questionnaire
TOS: The Obesity Society
TFEQ-R: Three Factor Eating Questionnaire, restraint factor of TFEQ
TFEQ-D: Three Factor Eating Questionnaire, disinhibition of control factor of TFEQ
TFEQ-H: Three Factor Eating Questionnaire, perceived hunger factor of TFEQ
USDA: United States Department of Agriculture
